# Proteomic Analysis of Pure Human Airway Gland Mucus Reveals a Large Component of Protective Proteins

**DOI:** 10.1371/journal.pone.0116756

**Published:** 2015-02-23

**Authors:** Nam Soo Joo, Idil Apak T. Evans, Hyung-Ju Cho, Il-Ho Park, John F. Engelhardt, Jeffrey J. Wine

**Affiliations:** 1 The Cystic Fibrosis Research Laboratory, Stanford University, Stanford, CA, 94305, United States of America; 2 Department of Anatomy and Cell Biology, Carver College of Medicine, University of Iowa, Iowa City, IA, 52242, United States of America; Pacific Northwest National Laboratory, UNITED STATES

## Abstract

Airway submucosal glands contribute to innate immunity and protect the lungs by secreting mucus, which is required for mucociliary clearance and which also contains antimicrobial, anti-inflammatory, anti-proteolytic and anti-oxidant proteins. We stimulated glands in tracheal trimmings from three lung donors and collected droplets of uncontaminated mucus as they formed at the gland orifices under an oil layer. We analyzed the mucus using liquid chromatography-tandem mass spectrometry (LC-MS/MS). Analysis identified 5486 peptides and 441 proteins from across the 3 samples (269–319 proteins per subject). We focused on 269 proteins common to at least 2 0f 3 subjects, of which 102 (38%) had protective or innate immunity functions. While many of these have long been known to play such roles, for many others their cellular protective functions have only recently been appreciated in addition to their well-studied biologic functions (e.g. annexins, apolipoproteins, gelsolin, hemoglobin, histones, keratins, and lumican). A minority of the identified proteins are known to be secreted via conventional exocytosis, suggesting that glandular secretion occurs via multiple mechanisms. Two of the observed protective proteins, major vault protein and prohibitin, have not been observed in fluid from human epithelial cultures or in fluid from nasal or bronchoalveolar lavage. Further proteomic analysis of pure gland mucus may help clarify how healthy airways maintain a sterile environment.

## Introduction

Human airways are constantly challenged by multiple viral, fungal, and bacterial pathogens. Airway innate defenses allow us to maintain near-sterile airways. The mechanisms include mucociliary and cough clearance, antimicrobial properties of airway mucus or ‘airway surface liquid’, and resident leukocytes [1,2,3]. When innate mucosal defenses are compromised, as in cystic fibrosis (CF) and chronic bronchitis [2,4], chronic respiratory bacterial and fungal infections occur, and when the adaptive immune system attempts to combat these, inflammation and tissue damage result.

Airway submucosal glands provide abundant mucus to the human upper airways [5]. The secretory part of the gland consists of two major cells types, the mucous cells, which secrete mainly mucin 5B (MUC5B), and the serous cells, which secrete electrolytes, water and a host of protective and innate defense proteins or “anti-proteins” because of their anti-microbial, anti-proteolytic, anti-oxidant, and anti-inflammatory properties. Serous cells have been described as “immobilized neutrophils” [1]. Serous cells express the cystic fibrosis transmembrane conductance regulator (CFTR) [6], a protein kinase A (PKA) and adenosine triphosphate (ATP)-activated anion channel that conducts chloride and bicarbonate across apical membranes. Loss of CFTR function leads to CF lung infections by compromising the multiple roles it plays in innate immunity [7,8,9,10], including glandular secretion [11,12,13,14,15,16,17,18].

Prior proteomic analyses of human bronchial alveolar lavage fluid/BALF [19,20,21,22,23,24,25], fluid from primary human tracheobronchial epithelial cell cultures [26,27] fluid from the Calu-3 cell line model of human airway gland serous cells [28], human nasal lavage fluid/NLF [29,30,31], and LC-MS/MS analyses of BALF from pigs [32] and mice [33] have been reported. The consensus from these studies is that airway and nasal fluids contain multiple proteins that help airways maintain near-sterility.

Because glandular mucus secretion is compromised in CF, it has been hypothesized that gland-derived innate defense molecules might be reduced in amount or bioavailability [3,10,34]. It is also possible that the profile of expressed proteins might become different as a result of the genetic change or secondarily as infection and inflammation proceed. A first step toward addressing this issue is to look at proteins in gland mucus that has not been mixed with the complex array of compounds secreted by surface epithelial and airway resident leukocytes. Here, we report the results of our attempts to do that. To obtain the purest samples of human submucosal mucus practicable, excised tracheal segments were stimulated with a mixture of forskolin and carbachol and mucus droplets were collected directly from gland orifices under oil. Under these conditions, the mucus doesn’t contact the epithelia surface, which has been cleaned and dried prior to applying the oil. The resulting mucus should contain only glandular secretions.

## Experimental Procedures

### Human airway tissues

All written consents from the donors were obtained for the use of tissue samples in the present study. The protocol for handling human airway tissues was approved (protocol#: 11638) by the Institutional Review Board of Stanford University, Stanford, CA 94305. Tracheal tissues utilized in the present study were obtained from tracheal trimmings (surgical scrap) from donor lungs at the time of transplant. Human airway tissues were placed in cold PhysioSol solution (Abbott Laboratories, IL, USA) and were used within 12 hours of the tissue acquisition.

### Glandular mucus collection

**Fig 1:**
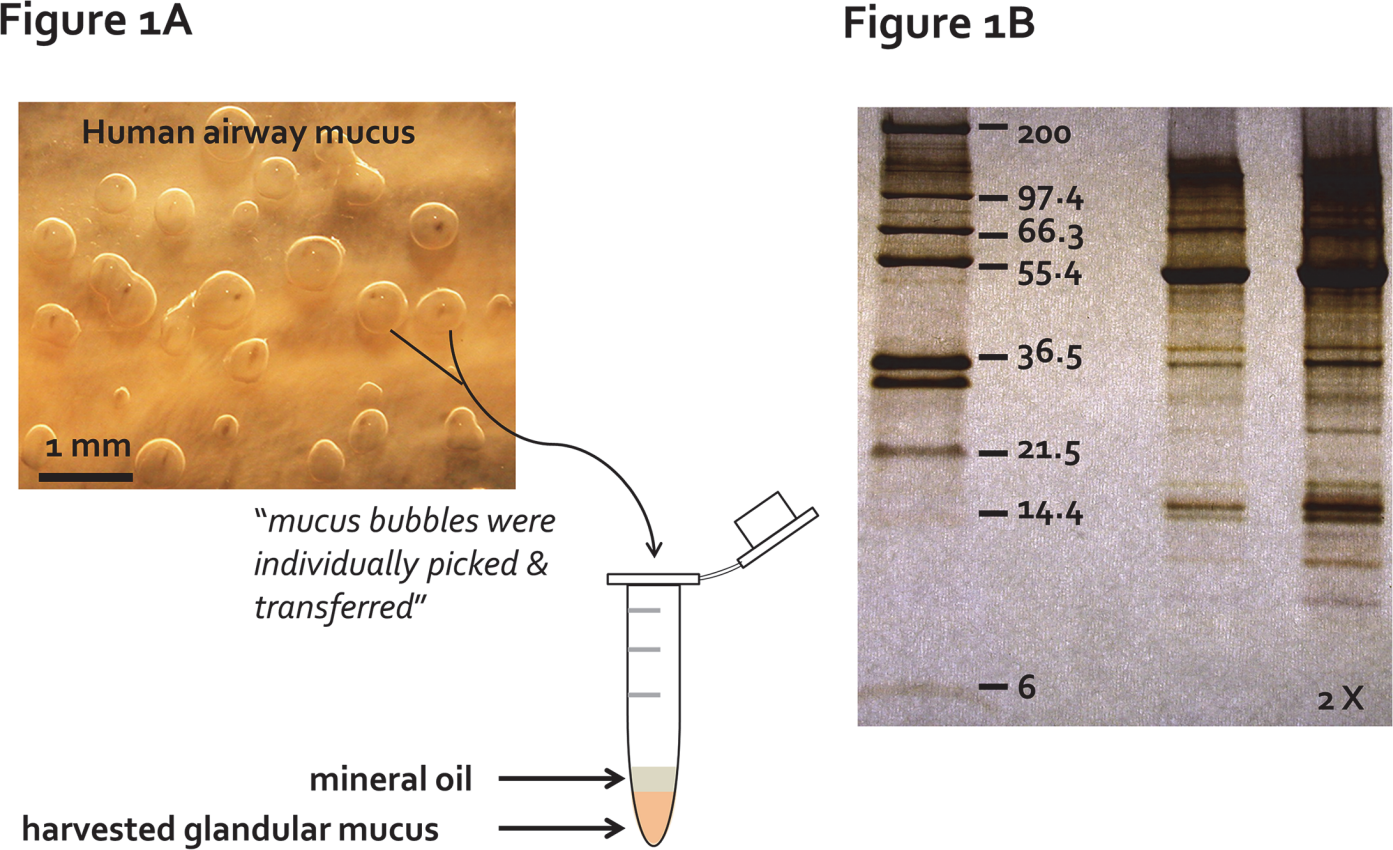
Primary pure human gland mucus collection. **(A)** Individual gland mucus bubbles, stimulated by a combination of 10 μM carbachol and 10 μM forskolin, formed under the water-saturated mineral oil on the tracheal mucosa, were picked up with sterilized microforceps and transferred into a sterilized Eppendorf tube for proteomics analysis. **(B)** A representative image of silver-stained protein bands from a human gland mucus sample collected as (A) on a Tris-Tricine 4–20% SDS polyacrylamide gradient gel. Protein molecular weight standard are shown on the left lane of the gel. Twice the same sample volumes were loaded on the far right lane.

Tracheal mucosae were dissected from the underlying cartilage in cold Krebs Ringer bicarbonate buffer oxygenated with 95% O_2_ and 5% CO_2_. The buffer composition was 115 mM NaCl, 2.4 mM K_2_HPO_4_, 0.4 mM KH_2_PO_4_, 25 mM NaHCO_3_, 1.2 mM MgCl_2_, 1.2 mM CaCl_2_, and 10 mM Glucose (pH 7.4 at 37°C) adjusted to ∼290 mosM with a Wescor vapor pressure osmometer (Logan, UT, USA). Indomethacin (1 μM) was present in the buffer solution to minimize endogenous prostaglandin production. Methods for agonist-stimulation were described previously [28,35]. Briefly a ∼1.5 cm^2^ tissue sample was mounted mucosal side in a 35 mm Petri dish lined with pliable silicone so that the glands were bathed in buffer while the surface was dry and could be covered with oil. Glandular secretion was stimulated with 10 μM forskolin + 10 μM carbachol (to achieve adequate amounts of mucus) and mucus bubbles that formed in the oil (**Fig. 1A**), collected individually using sterilized microforceps and transferred into a sterilized Eppendorf tube containing ∼50 μL water-saturated mineral oil. The collected mucus bubbles were stored at -20°C until use.

### Protein gels and liquid chromatography in-line with tandem mass spectrometry (LC-MS/MS)

Some of the mucus samples were run on Tris-Tricine 4–20% SDS polyacrylamide gradient gels followed by silver staining to separate protein bands (5∼200 kDa). Samples were diluted 1:10 with sample buffer containing 0.5% β-mercaptoethanol and incubated at 95°C at a Perkin Elmer thermocycler (Santa Clara, CA, USA) for 10 min. After gel-electrophoresis, the gel was exposed to Bio-Rad silver staining solution (Hercules, CA, USA) for 15–20 min.

Gland mucus samples from three healthy human donors (HN1-3) were sent to University of Iowa for LC-MS/MS experiments. The 3 donor subjects were male and 36.7 ± 14.8 (mean ± S.D.) years old. Water-saturated mineral oil in the gland mucus samples was removed by centrifugation. Each mucus sample was transferred to a new sterile Eppendorf tube and proteins were denatured in 100 μl of 50 mM Tris pH 8.5 buffer containing 8 M urea and 100 mM β-mercaptoethanol. Samples were lysed by passage through a 25 gauge needle using a 1 mL syringe and 50 μg total protein was prepared for MS analysis. Samples were reduced with 10 mM dithiothreitol for 1 hour at 37°C and then alkylated with 55 mM iodoacetamide for 1 hour at room temperature in the dark. After alkylation, samples were diluted with 50 mM Tris buffer pH 8.5 to have 0.5 M final urea concentration (1/16 dilution) and digested with trypsin overnight. Peptides were acidified with o-phosphoric acid and 1% acetonitrile was added. Samples were desalted using a C18 microspin cartridge (The Nest Group, MA, USA). Desalted samples were then applied to a SCX microspin cartridge (The Nest Group, MA, USA) and eluted using 4 different salt concentrations (20, 40, 60, and 120 mM KCl) according to manufacturer’s protocol. Eluted peptides were desalted and applied to an LC column in-line with the mass spectrometer. After separation of peptides, MS data were recorded on a LTQ-XL linear ion trap mass spectrometer (Thermo Fisher Scientific, MA, USA). A 90 minute gradient was applied for all the peptide separations on the LC column. Proteins were identified via two automated databases (a subset of the SwissProt 2012_09 and the SwissProt 2012_09 databases, 20,235 entries) and two search engines (Mascot Search Engine (version 2.4.0, Matrix Science, MA, USA) and X! Tandem (version cyclone, GPM)). The identified proteins by LC-MS/MS analysis with a minimum 2 peptides and 0% false discovery rate (FDR) were afforded by Mascot Search Engine. Maximum missed cleavages for trypsin were set to 2. Cysteine carbamodimethylation was used as fixed and methionine oxidation was used as variable modification. Mass tolerance for the parent ions was set to 1.8 Da and mass tolerance for fragment ions was set to 0.4 Da. The search results were compiled using Scaffold software (version 3.6.5, PS Inc., OR, USA). The settings of Scaffold were adjusted to 99.0% protein threshold and 90.0% peptide threshold. Therefore, all the proteins that are identified and presented in the manuscript have a False Discovery Rate (FDR) of 0% with 0 decoy proteins. To determine reproducibility, the same protein sample was analyzed 3x using the identical LC-MS/MS method. The initial run identified 68 proteins (100%), the second run, 62 proteins (91%) and the third run, 67 proteins (98%).

### Reagents

All reagents and drugs used in the present study were purchased from Sigma-Aldrich unless stated otherwise. Forskolin was dissolved in dimethyl sulfoxide, indomethacin in absolute ethanol and carbachol in sterile double distilled water. Aliquots of stock solutions were stored at -20°C and diluted to 1:1,000 with Krebs Ringer buffer solution before use.

## Results and Discussion

### Identification of proteins in pure human airway gland mucus

Mucus bubbles were collected as shown in **Fig. 1A**. To produce as much gland mucus as possible from the small tissue samples, we typically stimulated with forskolin + carbachol for 2–4 hours. This allowed collection of ∼10 μL of uncontaminated gland mucus. Our earlier study with porcine airways indicated that each agonist used alone produced mucus with comparable protein bands when run on SDS-PAGE with silver staining [28]. A representative example of human airway glandular mucus run on Tris-Tricine 4–20% gradient SDS-PAGE (**Fig. 1B**) shows more than 50 identifiable silver-stained protein bands.

**Fig 2:**
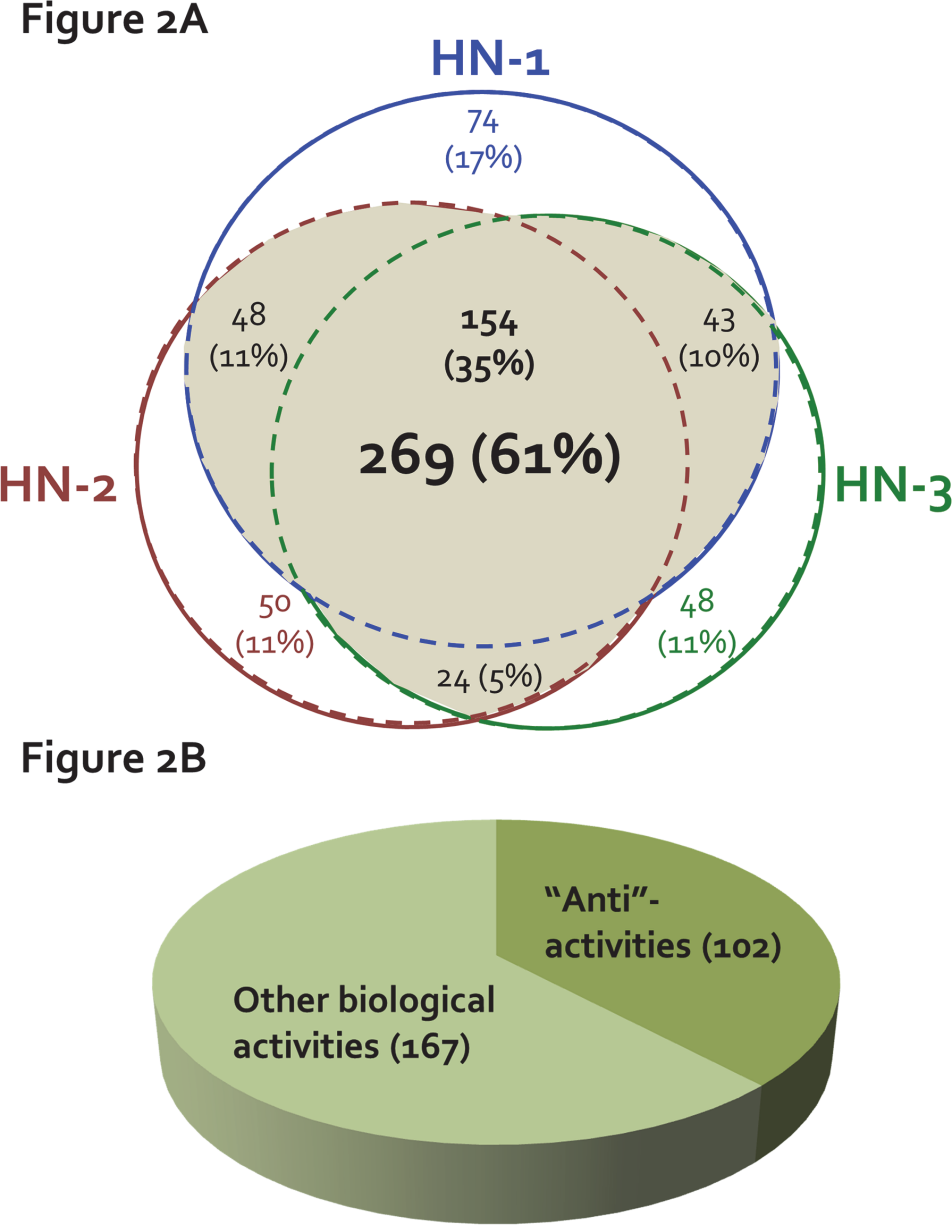
Proteins identified in human tracheal submucosal gland mucus by LC-MS/MS. **(A)** A modified Venn diagram of showing distribution of proteins among three healthy human (HN) donors. Across the 3 donors, 5486 peptides and 441 proteins were identified. Of these, 61% (269 proteins, the shaded area) and 35% (154 proteins) were found in 2–3 and all 3 HN gland mucus samples, respectively. **(B)** Among these 269 proteins, 102 (38%) were categorized as proteins with “anti”-activities: antimicrobial, antiinflammatory, antiproteolytic and antioxidant. Other biological activities include: biological regulation, metabolic/multi-organismal/developmental processes, binding activity, and establishment of localization.

By LC-MS/MS analysis, we have identified 5486 peptides (**S1 Table and S6 Table**) and 441 proteins (**S2 Table and S7 Table**): peptides: 2011, 1765 and 1710; proteins: 319, 276 and 269 from HN-1-3, respectively. As shown in **Fig. 2A**, 154 proteins (35%) were common to all three HN glandular mucus samples (**S3 Table**) and another 115 (26%) were present in 2 of 3 samples (**S4 Table**). Of these common proteins, 102/269 (38%) have been shown to have protective/innate immunity (**Fig. 2B**). Other overlapping biological activities of the 269 common proteins included (number of proteins): biological regulation (152); metabolic process (170); multicellular organismal process (118); developmental process (104); and establishment of localization (83) (**S5 Table**). In **Table 1**, the 102 proteins with protective roles were condensed into their respective families (e.g. 17 keratins represented by one family) even though not all members of a given family have been shown to play protective roles. **Table 1** includes proteins with well-established innate immune roles and others for which antimicrobial activity was established subsequent to the assignment of other functions. Eleven of these are highlighted below.

**Table 1:** Protein families with protective functions found in gland mucus.

Access #	MW	Identified Proteins	Access #	MW	Identified Proteins
P05091	56	Aldehyde dehydrogenase	P01876	38	Ig chains (5)
Q04828	37	Aldo-keto reductase	Q9Y6R7	572	IgGFc-binding protein
P02763	24	α-1-acid glycoprotein 1	P02538	60	Keratins (16)
P01011	48	α-1-antichymotrypsin	P02788	78	Lactotransferrin
P01009	47	α-1-antitrypsin	P30740	43	LE inhibitor
P07355	39	Annexins (5)	P51884	38	Lumican
P03973	14	Antileukoproteinase	P61626	17	Lysozyme C
P02647	31	Apolipoproteins (4)	Q14764	99	Major vault protein
P61769	14	β-2-microglobulin	Q9HC84	596	Mucins (2)
Q8TDL5	52	BPI fold-containing family A/B	P05164	84	Myeloperoxidase
P07339	45	Cathepsins (3)	P59665	10	Neutrophil defensin 1
P00450	122	Ceruloplasmin	P80188	23	NGAL-lipocalin
P01024	187	Complements (4)	Q06830	22	Peroxiredoxins (2)
P28838	56	Cytosol aminopeptidase	P01833	83	Polymeric Ig receptor
Q9UGM3	261	DMBT-1	Q99623	33	Prohibitins (2)
P07099	53	Epoxide hydrolase 1	P06454	12	Prothymosin alpha
P02671	95	Fibrinogen chains (3)	Q8IWL1	26	PS-A2
P21333	281	Filamins (2)	P08865	33	40S RP (7)
Q08380	65	Galectins/GBP (3)	P06703	10	S100-proteins (7)
P06396	86	Gelsolin	P02787	77	Serotransferrin
P09211	23	Glutathione S-transferase P	P29508	45	Serpin B3
P00738	45	Haptoglobin	P0DJI8	14	SA A-1 protein
P11142	71	HSC 71	P04179	25	Superoxide dismutase 2
P68871	16	Hemoglobin	P10599	12	Thioredoxin
P62805	11	Histones (2)	P25311	34	Zinc-α-2-glycoprotein
P30508	41	HLA class I antigen			

At least one member of these families of proteins with protective functions was found in 2–3 samples of human mucus, with (n) indicating the total number of family members found in human gland mucus. MW is shown as kDa. The full list of 102 protective proteins is found in S3 Table and S4 Table. Abbreviations: GBP, Galectin binding protein; DMBT-1, Deleted in malignant brain tumors 1 protein; HSC 71, Heat shock cognate 71 kDa protein; LE inhibitor, Leukocyte elastase inhibitor; PS-A2, pulmonary surfactant A2; 40S RP, 40S ribosomal proteins; and SA A-1, Serum amyloid A-1. Accession # is from the UniProt database http://www.uniprot.org/uniprot/.

### Examples of common gland proteins initially known for functions remote from innate defense

*Annexins* are cytosolic or membrane-attached proteins involved in vesicle transport during exocytosis and endocytosis, but annexins A1, A2, and A5 have been detected extracellularly [36]. An antifungal role has been demonstrated for annexin A1 [37] and annexin A1 is among the proteins whose expression is markedly increased in primary human airway epithelial cells exposed to *Pseudomonas aeruginosa* [38]. Annexin A2 helps to clear bacteria and viruses by facilitating their docking to macrophages and thus increasing macrophage phagocytosis [39].

*Apolipoproteins* are the major structural component of lipoproteins, which are mainly known for mediating lipid uptake and transport. However, apolipoproteins A1 and H also have anti-inflammatory and anti-oxidant activities [40], and apolipoprotein A1 is a also a potent anti-microbial against Gram positive *Staphylococcus epidermidis* [41]. Apolipoprotein H and peptides derived from it showed anti-microbial activity against both Gram positive *Staphylococcus aureus* and *S. pyogenes* and Gram negative *E. coli* [42].

*BPI (bactericidal/permeability-increasing) proteins*, also known as PLUNC (palate, lung and nasal epithelium clone) proteins are mainly expressed in exocrine glands of mouth, nose and the upper airways. Both BPI fold-containing protein family A1 (SPLUNC1, short PLUNC1) and family B1 (LPLUNC1, long PLUNC1) were detected in the HN1-3 gland mucus samples. BPI proteins have selective anti-microbial actions against Gram negative bacteria [43,44], and A1/SPLUNC1 has potent anti-proteolytic activity that endogenously regulates ENaC in airways [45] in a manner that should increase the volume of ASL.

*DMBT 1* (*deleted in malignant brain tumors 1 protein*) belongs to the scavenger receptor cysteine-rich, SRCR, superfamily [46] and the SRCR domain is known to bind various pathogens including *Streptococci, H. pylori* [47], and *HIV* [48]. The broad range of pathogen binding affinity of DMBT 1 allows it to serve as a pattern recognition molecule and to play a role in innate immunity. DMBT-1 can be found both in extracellular secretions and bound to the plasma membrane [49].

*Gelsolin* severs actin-filaments to aid actin assembly and disassembly. It is also found extracellularly, where it increases DNase-I activity by interfering with actin inhibition of DNase-1; resulting in a thinning of DNA-rich CF sputum [50]. A gelsolin-derived peptide, PBP-10, has antimicrobial activity against *E. coli, Pseudomonas aeruginosa*, and *S. pneumoniae* [51].

*Hemoglobin*, is known primarily for its role in red blood cells, and *haptoglobin is* known for its role in binding and clearing hemoglobin released from lysed red blood cells and is primarily expressed in hepatocytes, but both proteins are also expressed in other organs, including lungs [52,53]. Haptoglobin has an indirect anti-microbial effect in that it prevents free iron from being utilized by microbes [54,55]. It has long been known that hemoglobin has antimicrobial activity against Gram negative bacteria [56] and more recent work demonstrated broad antimicrobial activity of hemoglobin-derived peptides [57].

*Histones* are highly basic nuclear proteins and the principal structural component of chromatin. Their antimicrobial activity was accidentally discovered more than 5 decades ago during isolation of a bactericidal substance called phagocytin [58]. *Buforin I*, a well characterized toad stomach antimicrobial peptide, and *parasin I*, another bactericidal peptide, are derived from Histone 2A [59,60,61]. Histone 2A is also secreted into the amniotic fluid where it is antimicrobial and endotoxin neutralizing [62]. Histone H4 released from human sebaceous glands suppresses *Staphylococcus aureus* and *Propionibacterium acnes growth* [63].

*Keratins*, or *cytokeratins*, are fibrous structural proteins abundant in epithelial tissues and skin. Fourteen type I and II keratins, including keratin II 6A, constituted a large portion of structural proteins common to glands. Recently, Tom *et al*. discovered broad spectrum antimicrobial activity of glycine-rich C-terminal peptides from keratin 6A [64], with both *Staphylococcus aureus* and *Pseudomonas aeruginosa* being susceptible. Consistent with a role in innate immunity, multiple keratin genes in human primary airway epithelial cells show markedly increased expression after exposure to *Pseudomonas aeruginosa* [38]. It is unknown how many keratins and keratin-derived peptides have antimicrobial activity.

*Lumican* is an extracellular matrix proteoglycan, containing N-terminal leucine-rich domain, also found in skin, intestine and cornea. Lumican-deficient mice have impaired innate immunity when challenged with bacterial lipopolysaccharide [65]; they also exhibit defective clearance of *Pseudomonas aeruginosa* from the lung [66] and the cornea [67].

It has long been hypothesized that balanced activities of proteases and anti-proteases plays a key role in airway fluid and tissue homeostasis [16,28]. As shown in **S5 Table**, multiple anti-proteases, including α-1 antitrypsin, α-1 antichymotrypsin, antileukoproteinase, serpin, and leukocyte elastase inhibitor, and several proteases, including cathepsin, calpain and fibulin, were detected in at least 2 of 3 human gland mucus samples. The balance might be tipped toward proteolysis in CF because of abundant neutrophil-derived proteases coupled with reduced bioavailability of gland-derived anti-proteases.

### Origins of proteins in human airway submucosal gland mucus

Of the common 269 proteins, 194 (72%) are considered to be cytosolic/membrane proteins and 75 proteins (28%) are categorized as secreted proteins. Although great care was taken to insure that the collected mucus came directly from the duct orifices, the large number of proteins that are typically intracellular or membrane bound was unexpected and raises several possibilities.

First, although the mucus is ‘pure’ in the sense that it is only of glandular origin, it is not cell-free. Various types of cells have been observed in mucus while still within the gland ducts [68,69], and membrane proteins that are not in the apical membrane, such as Na^+^, K^+^, ATPase, were found in all samples. Because cells were expected to be a small component overall, we did not attempt to deplete them from the mucus for this initial proteomic analysis, but their presence needs to be kept in mind when considering the possible origins of the proteins.

A second possibility is that these cellular proteins are in the mucus because they have been secreted by pathways other than exocytosis. As discussed above, some of cellular proteins, e.g. keratins, histones, hemoglobin, peroxiredoxin and prohibitin have been identified previously in extracellular secretions, but because their secretion mechanisms are not understood they have not been classified as secreted proteins. In addition to exocytosis, at least four alternate means exist for exporting proteins: holocrine secretion, apocrine secretion, exosome release and microvesicle release. Holocrine secretion is poorly understood and in human seems to be limited to sebaceous and meibomian glands; recent evidence suggests that apoptosis may be the mechanism [70]. Apocrine secretion involves a portion of the apical membrane blebbing and then budding off, as in milk-protein secretion by the mammary gland [71]. Apocrine secretion has been observed in mouse airways [72,73,74] and may well occur in submucosal glands. In support of this hypothesis, CFTR-dependent secretion of mucus from skin glands of Xenopus laevis results in the loss of >75% of the mucous cell mass [75].

More recently, microvesicles (100–1,000 nm) budding from the plasma membrane and exosomes (40–100 nm) originating from multi-vesicular endosomes; have come to be viewed as important pathways by which nuclear, cytosolic, and membrane proteins are released into the extracellular environment [76,77,78,79,80,81]. Indeed, gland mucus imaged while still in the ducts has numerous particles in the size range of microvesicles as well as larger structures [82,83]. Surface proteins of secreted exosomes may serve as pathogen recognizing molecules, and exosome secretions are part of innate defense in primary human tracheobronchial epithelial cells [78], intestinal epithelial cells [79] and in salivary glands [81]. Based on the results of our present study and earlier investigations by others, a host of proteins that were considered to be exclusively intracellular until recently are in fact transported into airway mucus, where they or their peptide fragments participate in innate defense of the airways. We do not have a ready explanation for how the many cellular proteins have evolved additional mucosal defense functions. As more mucosal proteins are identified, it will be interesting to see if testable hypotheses on this point can be generated.

### A comparative analysis of innate defense and protective proteins of gland mucus with those of other airway fluids

To what extent does our sample of the human gland mucus innate defense proteome overlap that of other airway fluids? Data from LC-MS/MS analyses have been reported for nasal lavage fluid (NLF), apical fluid from primary human tracheobronchial epithelial cell culture (HTBE) [20,21,22,23,24,26,27,29,31] and bronchial alveolar lavage fluid (BALF) [20,21,22,23,24,26,27,29,31]. These proteomics studies of airway fluids have identified from 42 to > 1,500 proteins (n = 3∼18 healthy controls, BALF), 38 to 267 proteins (n = 6∼29 controls, NLF) and 132 to 186 proteins in apical fluid of primary human airway epithelial cultures.

For comparison we selected 35 protective proteins that were commonly detected in our 3 HN mucus samples **(Table 2)**. Among these, 18 were detected in all 4 sources of airway secretions (**Table 2**, top section) and the others were not reported for one or more sources. The largest overlap was with BALF, which is to be expected because BALF should include SMG mucus. Only two of the 35 proteins were absent from BALF: major vault protein (MVP) and prohibitin, both of which protect against bacterial infections in human and mice [84,85,86]. It is possible that these proteins were not detected in BALF because of the dilution factor. Sixteen of the proteins were not reported for apical fluid from airway cell cultures, which lack glands and also presumably lack proteins that might enter gland mucus via transcytosis or transudation, for example haptoglobin, immunoglobulin chains, and serotransferrin. Five proteins were not reported for nasal lavage fluid: heat-shock cognate 71 kDa protein, lumican, 40S-ribosomal protein SA, MVP and prohibitins. As with BALF, most of the NLF innate defense and protective proteins overlap SMG proteins. Nasal submucosal glands and anterior nasal glands are likely the major sources of these proteins.

**Table 2:** A comparative analysis of anti-proteins of 4 airway secretions.

Identified Proteins	SMG	BALF	HTBE	NLF
Alpha-1-antichymotrypsin	**√**	**√**	**√**	**√**
Alpha-1-antitrypsin	**√**	**√**	**√**	**√**
Annexins	**√**	**√**	**√**	**√**
Antileukoproteinase	**√**	**√**	**√**	**√**
Apolipoproteins	**√**	**√**	**√**	**√**
BPI fold-containing family A/B (PLUNC)	**√**	**√**	**√**	**√**
Ceruloplasmin	**√**	**√**	**√**	**√**
Complements	**√**	**√**	**√**	**√**
Deleted in malignant brain tumors 1 protein	**√**	**√**	**√**	**√**
Fibrinogen	**√**	**√**	**√**	**√**
Hemoglobin	**√**	**√**	**√**	**√**
Histones	**√**	**√**	**√**	**√**
Keratins	**√**	**√**	**√**	**√**
Lactotransferrin	**√**	**√**	**√**	**√**
Mucin-5B	**√**	**√**	**√**	**√**
Neutrophil gelatinase-associated lipocalin	**√**	**√**	**√**	**√**
Polymeric immunoglobulin receptor	**√**	**√**	**√**	**√**
Cathepsin D	**√**	**√**	**√**	**√**
Galectin-3-binding protein	**√**	**√**	**√**	**√**
Gelsolin	**√**	**√**	**√**	**√**
Glutathione S-transferase P	**√**	**√**	**√**	**√**
Peroxiredoxins	**√**	**√**	**√**	**√**
Serpin B3	**√**	**√**	**√**	**√**
Thioredoxin	**√**	**√**	**√**	**√**
Zinc-alpha-2-glycoprotein	**√**	**√**	**√**	**√**
Heat shock cognate 71 kDa protein	**√**	**√**	**√**	
Haptoglobin	**√**	**√**		**√**
Ig chains	**√**	**√**		**√**
Alpha-1-acid glycoprotein 1	**√**	**√**		**√**
IgGFc-binding protein	**√**	**√**		**√**
Serotransferrin	**√**	**√**		**√**
Lumican	**√**	**√**		
40S ribosomal protein SA	**√**	**√**		
Major vault protein	**√**			
Prohibitins	**√**			

The data represent a summary of our comparative analysis of 35 common protective HN anti-proteins in the present study to 3 other airway fluids. Abbreviations: SMG, airway submucosal gland mucus; BALF, bronchial alveolar lavage fluid; HTBE, apical fluid of primary human tracheobronchial epithelial cell culture; and NLF, nasal lavage fluid. The data from other airway fluids are referenced in the text.

### Limitations of this study

The small number of subjects and the limited amount of uncontaminated gland mucus that could be collected from each one will bias toward an underestimate of the gland mucus proteome—especially for low molecular weight proteins. Because we were stimulating the glands *ex vivo*, we would also underestimate proteins that are transcytosed from the blood. Conversely, we did not attempt to deplete resident cells from the mucus and as discussed, these probably contributed to the proteins we detected. If these are the cells that are normally found in glands, for example leukocytes that enter the airways via the glands, their inclusion may be informative. However, we used a high concentration of secretory agonists to insure adequate samples of mucus, which, together with unintended damage from dissection and an unavoidable level of hypoxia in *ex vivo* tissues, could have led to higher than normal levels of epithelia sloughing.

### Summary and conclusions

Airway diseases such as asthma, chronic obstructive pulmonary disease and CF are associated with chronic airway inflammation and airway infections are either chronic or more frequent. People with CF usually die from chronic bacterial lung infections. These infections are localized to the mucus layer of the lung and rarely cause sepsis. Although CF is caused by loss of function of a single anion channel, CFTR, but that loss leads to multiple defects in mucosal defenses [8,14,87,88]. We hypothesized a role for airway glands in innate host defense, based on the recognized role of mucus clearance in protecting the airways [2] and the glandular source of much mucus [5]. Because glands express CFTR [6], we sought and found evidence that gland secretion is defective in CF [3], and suggested this was one reason why CF lungs are vulnerable to infections. This study supports those hypotheses by showing that a substantial proportion of the proteins in human airway gland mucus have protective functions.

The present study suggests two ways in which mucus is protective beyond its critical role in mechanical clearance [2]. First, in direct support of physical entrapment and removal of pathogens, mucus has a host of anti-microbial compounds and proteins that kill pathogens or inhibit their growth. Importantly, these anti-microbial mechanisms are extremely diverse and sometimes synergistic, which should make it more challenging for resistance to develop. Second, to deal with toxic compounds that are either inhaled or generated to confront pathogens, mucus also contains a host of anti-proteolytic, anti-oxidant, or anti-inflammatory proteins that help protect airway tissue. Finally, although not directly studied here, mucus contains cells like neutrophils and macrophages that kill pathogens with yet another set of overlapping mechanisms.

The sophisticated orchestration the innate mucosal defenses contrast in two fundamental ways with the use of synthetic antibiotics in most clinical settings. First, as the term ‘innate’ emphasizes, these defenses are ever present in the airways, ready to act as soon as a single pathogen makes contact with the mucus, rather than after infections have become established. Second, as emphasized above, our natural defenses are multi-factorial, unlike the mono-antibiotic therapies that still dominate clinical practice.

To conclude, we established the feasibility of carrying out a proteomics analysis of ‘pure’ human airway gland mucus, and showed that a large proportion of the proteins in mucus have protective roles. Many of the proteins encountered have traditionally been considered to play exclusively intracellular roles, but it is increasingly clear that cells can export these proteins to the extracellular space, where they or their peptide fragments take on new roles. It seems likely that additional protective roles will be uncovered for many of the other proteins that are resident in airway mucus.

## Supporting Information

S1 Table Total peptide fragments found in 3 HN mucus samples. Peptides (and proteins in S 2) were identified via two databases, a subset of the *SwissProt* 2012_09 and the *SwissProt* 2012_09 databases with 20,235 entries, and two search engines, *Mascot Search Engine* and *X*! *Tandem*. The detailed set up for the search engines: Fragment Tolerance: 0.40 Da (monoisotopic), Parent Tolerance: 1.8 Da (monoisotopic), Fixed Modifications: +57 on C (carbamidomethyl), Variable Modifications: +16 on M (oxidation), and Digestion Enzyme: trypsin. The peptide thresholds of *Scaffold* software were adjusted to 90% minimum.
(XLSX)

S2 Table Unique peptides observed for each HN sample and relative abundance of 441 HN proteins. The peptide thresholds of *Scaffold* software were adjusted to 99% minimum and two peptides minimum. The 441 HN proteins were arranged as a rank order of their relative abundance (#1, the most abundantly detected), based on Total Ion Current (TIC) values of individual proteins calculated by *Scaffold* after normalizing the dataset for TIC.
(XLSX)

S3 Table Proteins found in 3 of 3 HN mucus samples, top 65 proteins have protective properties. The protective and the other group proteins (total 154 proteins) are arranged according to their relative abundance (RA), from S2 Table, in each group.
(XLSX)

S4 Table Proteins found in 2 of 3 HN mucus samples, top 37 proteins have protective properties. Additional 114 proteins detected in 2 of 3 HN samples, other than the ones listed in S3 Table, are arranged according to their relative abundance (RA) in each group.
(XLSX)

S5 Table Biological activities of 269 proteins found in at least 2 of 3 HN mucus samples. The list of 269 proteins is arranged according to their relative abundance.
(XLSX)

S6 Table HN peptides with additional MS/MS information. (XLSX)

S7 Table HN Proteins with additional MS/MS information. (XLSX)
